# The Impact of Age, Gender, Socioeconomic Status, and Education Level on Influenza Vaccination Hesitancy in the Henry Ford Family Health Center Patient Population

**DOI:** 10.51894/001c.164363

**Published:** 2026-07-31

**Authors:** Shumail M. Rathore, Scott Nyman, Samuel J. Wisniewski, Welter Hope

**Affiliations:** 1 Henry Ford Genesys Hospital

## 35

### Background

Influenza vaccination is a cornerstone of preventive care and a key strategy for reducing influenza-related morbidity and mortality. Despite longstanding public health recommendations, adult influenza vaccination rates remain suboptimal, particularly in primary care populations. Vaccine hesitancy is influenced by a complex interplay of demographic, socioeconomic, and belief-based factors.

### Methods

This mixed-methods observational study included a retrospective chart review (N = 155) and an anonymous cross-sectional patient survey (N = 168) conducted at the Henry Ford Family Health Center–Burton. Descriptive statistics were used to characterize the study population. Inferential analyses, including chi-square testing and binomial logistic regression, were performed to evaluate predictors of influenza vaccination.

### Results

Influenza vaccination rates were low in both cohorts, with 19.0% of survey respondents and 25.8% of patients identified through chart review documented as vaccinated. Regression analysis of chart review data did not identify significant demographic predictors. In contrast, survey-based regression analysis was statistically significant (*p* < 0.01), demonstrating that increasing age (OR 1.03) and attainment of a high school diploma or GED (OR 3.25) were associated with higher odds of vaccination. Vaccine hesitancy was driven primarily by low perceived risk of influenza, fear of side effects, and missed opportunities, rather than distrust of healthcare providers or social media influence.

### Conclusion

Influenza vaccination uptake in this Family Medicine clinic population is shaped largely by modifiable factors, including education level, risk perception, and provider recommendation. These findings highlight actionable targets for clinic-based interventions aimed at improving vaccination rates.

